# A combination of logical judging circuit and water-resistant ultrathin film PEDOT: PSS electrode for noninvasive ECG measurement

**DOI:** 10.1186/s11671-024-03988-9

**Published:** 2024-03-14

**Authors:** Kewei Song, Kayo Hirose, Kioto Niitsu, Tsubasa Sui, Hiroto Kojima, Toshinori Fujie, Shinjiro Umezu

**Affiliations:** 1 Department of Modern Mechanical Engineering, Graduate School of Creative Science and Engineering, Waseda University, 3-4-1 Okubo, Shinjuku-ku, Tokyo, 169-8555 Japan; 2 Anesthesiology and Pain Relief Center, The University of Tokyo Hospital, 7-3-1 Hongo, Bunkyo-ku, Tokyo, 113-8655 Japan; 3 Department of Integrative Bioscience and Biomedical Engineering, Graduate School of Advanced Science and Engineering, Waseda University, 3-4-1 Okubo, Shinjuku-ku, Tokyo, 169-8555 Japan; 4 Department of Modern Mechanical Engineering, Waseda University, 3-4-1 Okubo, Shinjuku-ku, Tokyo, 169-8555 Japan; 5 School of Life Science and Technology, Tokyo Institute of Technology, B-50, 4259 Nagatsuta-Cho, Midori-ku, Yokohama, 226-8501 Japan

**Keywords:** PEDOT: PSS, Water resistance, Non-adhesive, ECG monitoring, Logical judging circuit

## Abstract

Heart disease-related deaths have increased in recent decades, with most patients dying of sudden cardiac arrest. In such instances, the effect of regular electrocardiogram (ECG) measurements is minimal. Therefore, long-term ECG monitoring has become increasingly important. In this paper, we report a non-adhesive high accuracy ECG monitoring system that can be used in various scenarios without interfering with daily activities. The ECG ultra-thin film electrode is made by water-resistant material based on poly(3,4-ethylenedioxythiophene) poly(4-styrenesulfonate) (PEDOT: PSS) electrode doped with ethylene glycol (EG) and xylitol, to improve the noise signal caused by sweat. The optimal ratio of the three ingredients of PEDOT: PSS/xylitol/EG was determined experimentally to accommodate the ECG monitoring. By using the proposed selectively closed multi-channel single-lead logic circuit, the noise of ECG signal received from the proposed film electrode can be successfully reduced during broad-area electrode measurements, thus to improve ECG measurement accuracy.

**Supplementary Information:**

The online version contains supplementary material available at 10.1186/s11671-024-03988-9.

## Introduction

According to a latest World Health Organization study, noncommunicable diseases including heart disease now account for seven of the top ten causes of death worldwide [1]. In recent years, the number of deaths due to heart disease has continued to account for a high proportion of deaths due to noncommunicable diseases or accidents. Furthermore, according to a survey conducted by the Ministry of Health, Labor, and Welfare, the average life expectancy and healthy life expectancy of Japanese citizens are increasing, but the proportion of deaths due to heart disease has increased to approximately 15% of all deaths in the country, second only to tumors and cancer, with the number of deaths rising from 146,171 in 2000 to 207,714 in 2019 [2]. A considerable percentage of deaths from heart disease are due to lethal arrhythmias, and these patients are usually undiagnosed or previously classified as having a low cardiovascular risk [3]. In addition, the virus that caused the new coronary epidemic in the last two years, i.e., severe acute respiratory syndrome-coronavirus-2 (SARS-CoV-2), can use angiotensin-converting enzyme 2 (ACE2) as a functional receptor for cell entry, implying that SARS-CoV-2 may use this entry pathway to invade and directly damage cardiomyocytes [4]. Other related reports have indicated that cardiac injury combined with COVID-19 can have more detrimental consequences [5–8]. Therefore, long-term ECG monitoring of potential cardiac patients is necessary to confirm their heart status as soon and as accurately as possible.

One of the most common techniques for long-term ECG monitoring is an implantable loop recorder (ILR), which is typically used in clinical applications. However, its use requires costly surgical procedures and poses an infection risk [9], making it less preferable. In addition, other standard ECG measurement devices, such as the Holter Monitor, cannot be considered as the first option for long-term monitoring. Although they can provide high-quality ECG signals, they can be used continuously only for 24–48 h. These devices require cables that obstruct the user's daily activities, skin adhesive-based electrodes which may cause patient discomfort after a few days [10], and glue that degrades with time, allowing the electrodes to slip off owing to sweating. Recently, there have been some reports on E-textiles, a wearable ECG measurement device that is worn on the body and acquires waveforms comparable to those obtained by a Holter Monitor [11]. However, compared to the Holter Monitor, the ECG monitoring accuracy using E-textiles still requires improvement [12–16].

Apple Inc. has launched the Apple Watch series, which, after the third generation, allows users to record heart rhythm strips and aids in the self-diagnosis of atrial fibrillation (AF). The capacity of this algorithm to detect AF has been approved by the US Food and Drug Administration [17–19].

Samsung Inc. has also launched a smartwatch series that includes ECG monitoring. However, as we can see from related reports, ECG data obtained by smartwatches are calculated using an algorithm. Therefore, the accuracy is highly dependent on the algorithm and must be optimized [20].

Other studies have reported unbonded long-term monitoring methods [21–23]. Zhongjie et al. demonstrated an ECG measurement method using multiple unipolar capacitively coupled electrodes attached to the backrest and seat surfaces of a chair [24]. This report demonstrates the feasibility of non-adhesive ECG measurements. However, because multiple electrodes resulted in a wider contact area, the friction and sweat of the subjects caused more significant noise in the ECG images.

Among all the measuring instruments introduced above, the wet Ag/AgCl electrode is the most commonly used in ECG measuring instruments. However, long-term monitoring poses a significant risk of skin irritation, inflammation, and allergic responses [25–27]. Moreover, the signal quality deteriorates as the gel dehydrates. For a dry Ag/AgCl electrode, it is not necessary to consider that the signal quality decreases over time. However, a dry Ag/AgCl clip electrode must be attached to the subject, which significantly impacts the subject’s daily activities [28]. Thus, it cannot be used for long-term measurement of those classified as low risk.

**Fig. 1:**
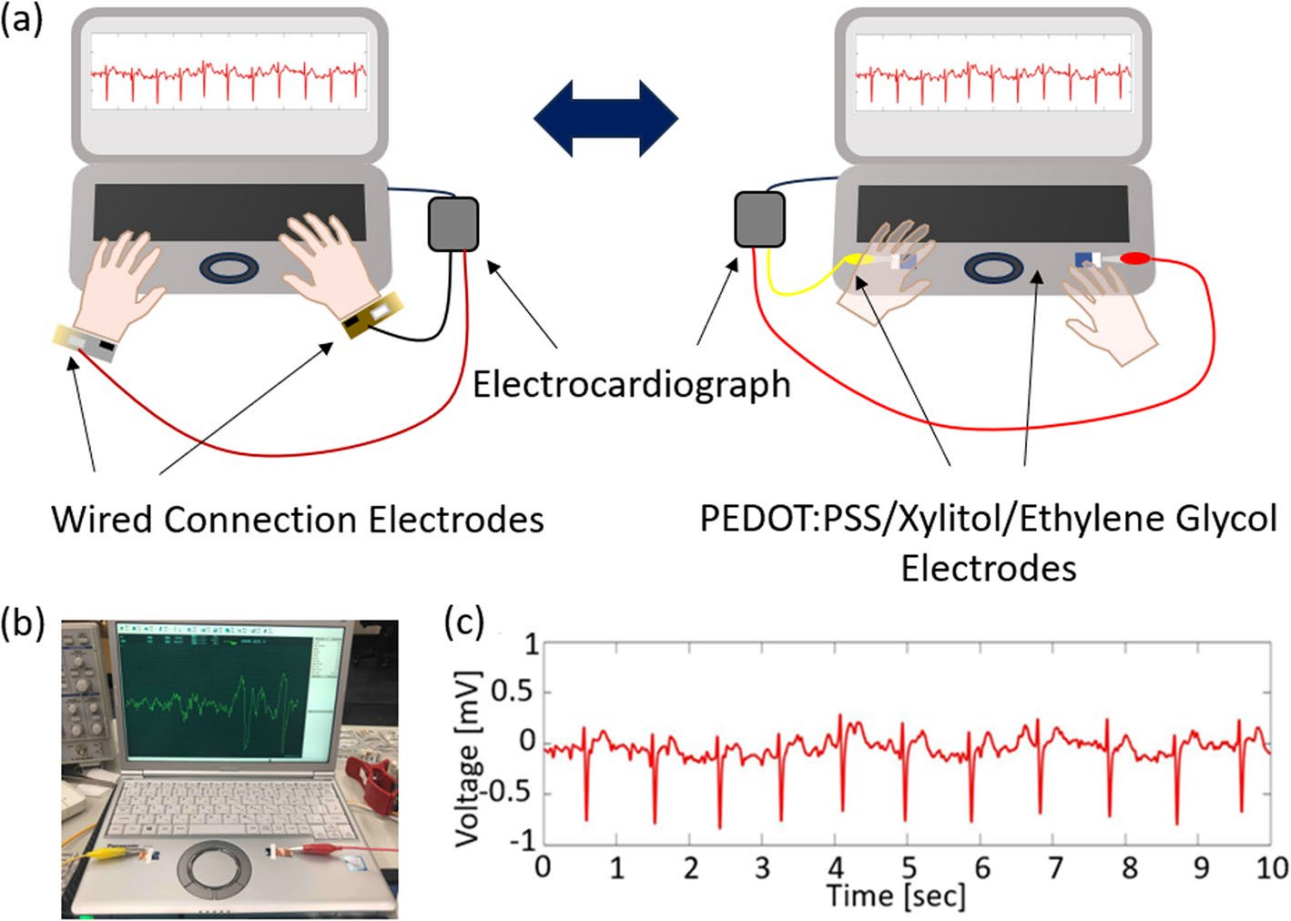
**a** Schematic illustration of the conventional adhesive ECG measurement device and our designed non-adhesive ECG measurement device for daily monitoring; **b** Photo illustration of the ECG monitoring electrode setup on a laptop; **c** ECG measured using the 3.5 wt% EG/5 wt% xylitol/PEDOT: PSS electrode on the laptop. While slight noise is superimposed on the baseline, the primary P-QRS-T wave is present in the electrocardiogram

Recently, many studies have been conducted to replace Ag/AgCl electrodes. Huang et al. [29] developed a sponge ECG electrode for long-term monitoring that includes a gel-free silver nanowire (AgNW)/polyvinyl butyral (PVB)/hydrophilic polyurethane (PU) sponge. Nishat et al. [30] demonstrated a combination of multiwalled carbon nanotube and dimethylpolysiloxane (PDMS), which were deposited on a Kapton substrate and silver. Dong et al. [31] reported the fabrication of acetylene carbon black/polydimethylsiloxane (PDMS) electrodes using a two-step process. However, these electrodes generally require complicated and expensive fabrication processes, making their replication and commercial application more difficult. Although they are dry electrodes, they still require the subject to attach them to the skin for an extended period, which unavoidably causes discomfort, as shown in Fig. 1a. In addition, because the ECG signal depends on the placement and position of the electrodes, movement of the subject may introduce noise. Therefore, electrodes for long-term monitoring must be flexible, lightweight, and highly sensitive. Compared with other electrodes, PEDOT: PSS has been extensively studied in the bioelectronics field in recent years because of its flexibility, biocompatibility, and ease of processing [32–35]. However, compared with metallic electrodes, the electrical conductivity of PEDOT: PSS thin film remains low. It is also challenging to use in long-term monitoring and preservation because of water dispersion features. Focusing on these limitations, many studies have shown that adding appropriate additives to PEDOT: PSS is effective [36–40]. Okuzaki et al. and Lin et al. [41, 42] demonstrated that EG can significantly improve the conductivity of PEDOT: PSS. As a widely used solvent for PEDOT:PSS to increase conductivity, EG can improve the water-resistance characteristic, and has a better biocompatibility characteristic among the other chemicals such as DMSO [43–45]. In addition, xylitol has been reported as a feasible additive for promoting the water resistance of PEDOT: PSS [46]. However, studies integrating these two additives have not yet been reported.

**Fig. 2:**
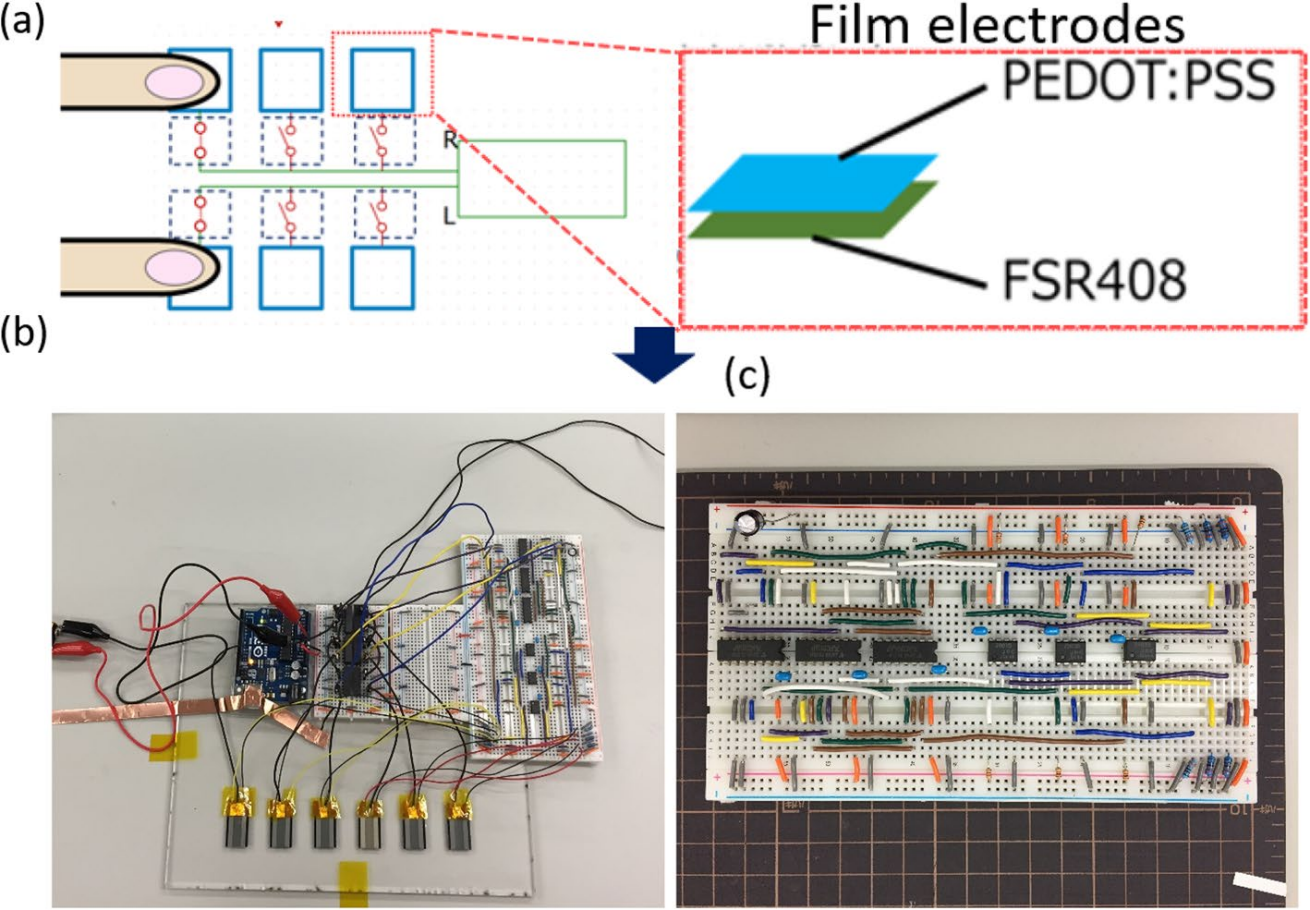
**a** Schematic illustration of the combination of the thin-film electrodes and the logical judgment circuit; **b** Actual electrodes and logical judgment circuit; **c** Logical judgment circuit constructed on a breadboard

Herein, we developed a non-adhesive ECG monitoring system that can be used in various scenarios without interfering with daily activities, as shown in Fig. 1a, b. The electrodes designed in this study were made of a PEDOT: PSS/xylitol/EG composite, which is water-resistant, ultrathin, and can generate clear ECG signals, as shown in Fig. 1b, c. We evaluated the effects of different concentrations of xylitol in a PEDOT: PSS precursor on the water resistance and discovered that the sample with a concentration of 5 wt% xylitol exhibited the best water resistance. Subsequently, based on the improvement in the water resistance, we added varying concentrations of EG to the PEDOT: PSS/xylitol composite to improve its conductivity so that the electrodes could be more sensitive. Regarding to the interfering signals generated by poor contact between the composite and the human body, the study used the signal-to-noise ratio (SNR) algorithm, a widely used algorithm for filtering ECG signals, to analyze the acquired ECG signals [47, 48]. Furthermore, because non-adhesive monitoring requires more electrodes than standard monitoring, the contact area expands, introducing additional inaccuracies into the ECG waveforms. To ensure its accuracy, we designed a logical judging circuit (Fig. 2a) that could selectively close the testing channel and reduce unnecessary contact area using the integrated circuit unit, as shown in Fig. 2b, while Fig. 2c shows the complex filter circuit we made on a breadboard.

## Method

### PEDOT: PSS thin-film electrode fabrication

For the water-resistant films, we added xylitol (Sigma-Aldrich) and dimethyl sulfoxide (DMSO) (Sigma-Aldrich) to PEDOT: PSS (Sigma-Aldrich, St. Louis, MO) and investigated the effects of these two additives on the water resistance of the thin-film electrodes. The thin-film electrode fabrication process followed that described by Greco et al. [49]. The specific practical steps were as follows. First, the case was filled with a polyvinyl alcohol (PVA) (Fujifilm Wako Chemicals Corporation, Japan) solution adjusted to 10 wt%. After a dip in the paper substrate (20 mm × 20 mm), it was spin-coated as a sacrificial layer at 4000 rpm for 20 s and dried at 100 °C for 1 h to evaporate water. After that, a polydimethylsiloxane (PDMS) (Sylgard 184, Dow Corning Company, Midland, MI) solution was spin-coated at 2000 rpm for 2 min and baked at 100 °C for 1 h. Subsequently, the surface of PDMS was modified to become hydrophilic by UV ozone cleaning (ASM401N OZ, Asumi Giken, LTD., Japan) for 1 h, and then PEDOT: PSS with 5 wt% DMSO or 5 wt% xylitol was deposited on the PDMS by spin coating at 1000 rpm for 2 min on the treated devices. After that, the samples were heated at 140 °C for 15 min for DMSO and 120 °C for 12 h in xylitol. Finally, the PEDOT: PSS/PDMS ultrathin electrode was obtained by placing the glass substrate in water and dissolving the sacrificial layer of PVA.

Based on the outcome of the water-resistance experiment, we added EG to the PEDOT: PSS/5 wt% xylitol composite film to increase conductivity. The EG concentrations were divided into seven groups. The concentration increment was 0.5 wt%, from 2 to 5 wt%. The impedance of each group was measured using a four-point probe measurement method, with water as the medium and glass as the substrate, completed a TER-2000RH type impedance measurement device (ULVAC Riko Co., Ltd.). The deposition process was the same as that used for PEDOT: PSS/5 wt% xylitol.

**Fig. 3:**
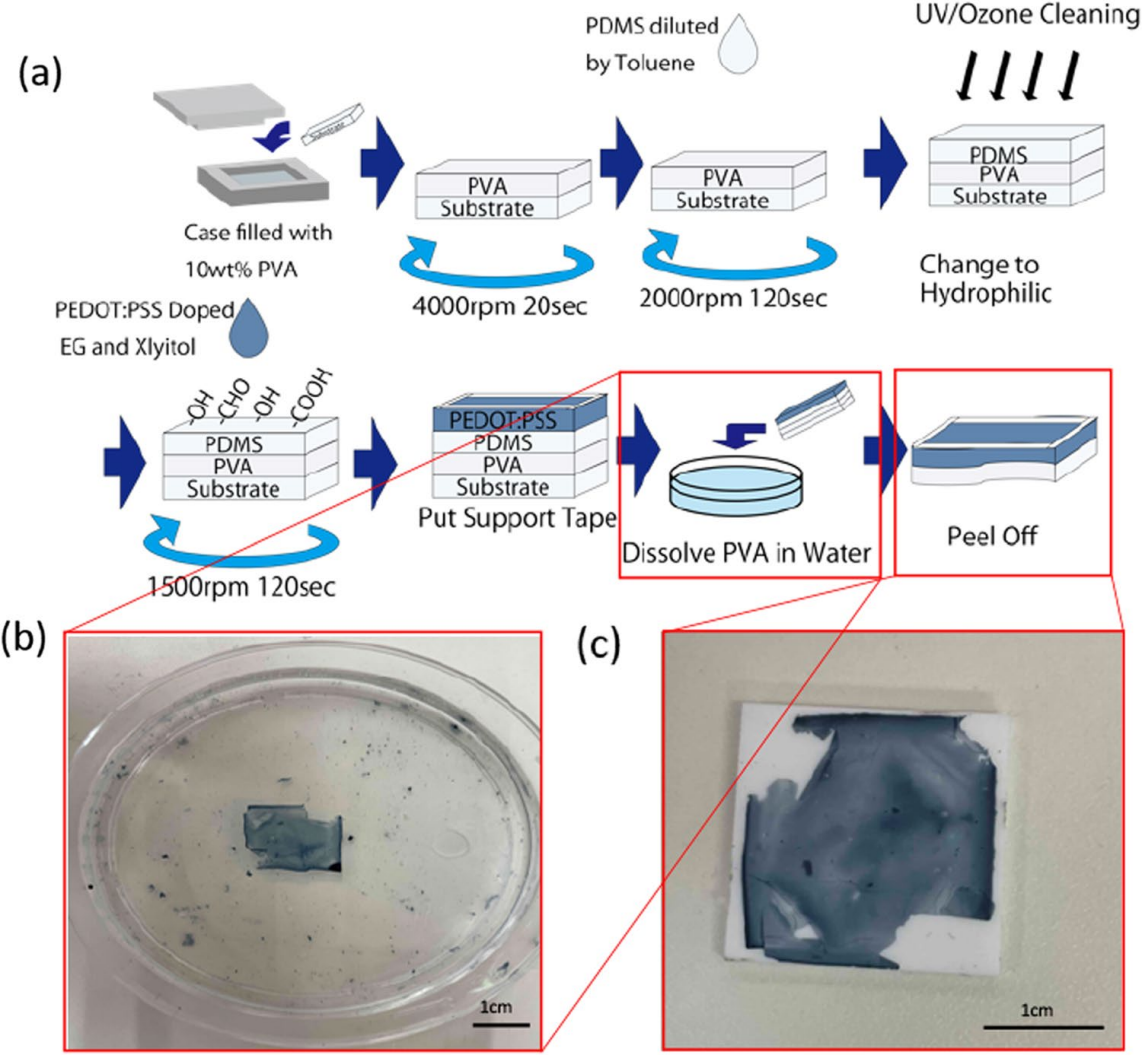
**a** Schematic illustration of the EG/xylitol/PEDOT: PSS thin-film electrode fabrication process, using paper as the substrate and PVA as a sacrificial layer. Water osmotic peeling is performed instead of manual thin-film recovery, and. support tapes placed around the substrate facilitate handling in water; **b** Photo of separation of the thin-film electrode; **c** The recovered EG/xylitol/PEDOT: PSS thin-film electrode after separating from the substrate

Furthermore, to optimize the thin-film separation process, we investigated the optimal thickness of the support layer. The specific experimental steps are as follows. The first step is similar to the previously mentioned process. We put the paper substrate (20 mm × 20 mm) in a case filled with 10 wt% PVA solution and then spin-coated the substrate at 4000 rpm for 20 s and dried at 100 °C for 1 h to obtain the PVA sacrificial layer. PDMS with different ratios of toluene (Sigma-Aldrich, St. Louis, MO) (PDMS:toluene v/v 10/1, 1/1, 1/2, 1/4) was then deposited on the substrate/PVA by spin coating at 2000 rpm for 2 min and baked at 100 °C for 1 h. Subsequently, the PEDOT: PSS layer was deposited on the devices and automatic separation was performed as previously mentioned. A schematic illustration of the fabrication process and separated thin-film electrode is shown in Fig. 3a–c.

### Evaluating the performance of the PEDOT: PSS electrode

We evaluated the performance of the fabricated electrode by analyzing the ECG signal to calculate the signal-to-noise ratio (SNR) using the following equation [50, 51]:

$$SNR = 20\log (S/(S^{\prime } - S)),$$

where S is the signal filtered at frequencies ranging from 0.5 to 100 Hz, and S’ is the ECG signal without filtering.

### Logical judging circuit design

**Fig. 4:**
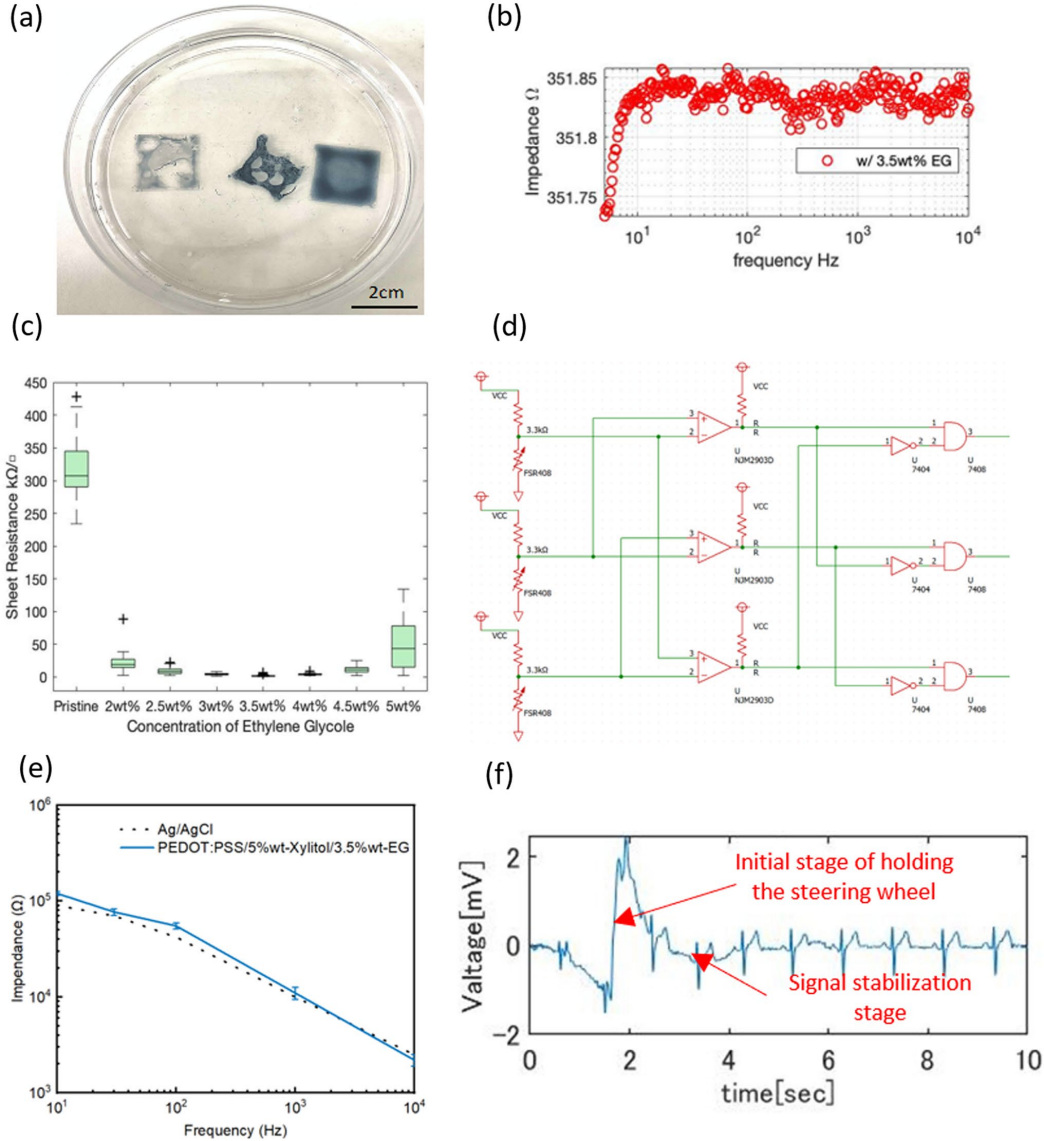
**a** The thin-film electrodes after soaking in water for 24 h, starting from the left is pristine, 5 wt% DMSO added, and 5 wt% xylitol added PEDOT: PSS thin-film; **b** Impedance figure of the 3.5 wt% EG/5 wt% xylitol/PEDOT: PSS thin-film electrode, average 351 **Ω**; **c** Sheet resistance of EG/5 wt% xylitol/PEDOT: PSS thin-film electrode, the concentration of EG varied from 2 to 5 wt%; **d** Schematic illustration of the logical judgment circuit design. The setting condition is to check each electrode’s voltage from the first on the right/left, and only connect the electrode with the highest pressure; **e** Comparison of skin–electrode impedance spectra for the proposed electrode and commercial Ag/AgCl electrode; **f** ECG signal for a person holding a steering wheel for a very short time; the signal is detected stably and accurately

To ensure the accuracy of the non-adhesive ECG monitoring developed in this study, we divided the large-area electrodes into several smaller electrodes. Thus, more signal input points were generated. The design of the logical judging circuit enables the automatic judgment of the available channel for receiving ECG signals by determining the input point under the highest pressure. In this situation, the other signal channels are temporarily shut down to reduce the noise. In this experiment, we prepared six electrodes for ECG measurements. The electrodes were made of PEDOT: PSS films, and each electrode was on an FSR408 pressure sensor, connected in series. The electrodes were connected to the R- and L-terminals of an ECG Explorer 500X2 (San-Ei Medisys Co., Ltd., Japan) via a relay. Because this pressure sensor has a lower resistance under higher pressure, the electrode under the highest pressure will have the minimum voltage. The setting condition was to check the voltage of each electrode from right to left, and only the electrode with the highest pressure would be connected. The other channels are shut down. The circuit diagram is shown in Fig. 4d. Please refer to the Supplementary Materials for the design of the filters and amplifiers (Figs. S2–S4) and testing process (Figs. S5–S7).

## Results and discussion

### Water resistance

To evaluate methods of improving water resistance of the thin film, the sample with DMSO added was heated for 15 min at 140 °C, and the sample with xylitol added was heated for 12 h at 120 °C. Thereafter, we placed the samples in water for 24 h. The results are shown in Fig. 4a. Starting from the left the samples are the pristine PEDOT: PSS thin film without any dopant, the sample with 5 wt% DMSO, and the sample with 5 wt% xylitol added. Although the PDMS support layer of the pristine sample was treated with UV ozone cleaning to make it hydrophilic, significant surface dispersion (destruction) was still observed after 24 h in water. In the case of the DMSO-added sample, dispersion was not apparent; however, the conductive layer spontaneously peeled off. On the sample containing xylitol, no dispersion or peeling of the conductive layer was observed after 24 h in water. The results of the experiment were consistent with those reported by Li et al. [52], who found that xylitol has two functions in improving stability: as a plasticizer preventing hydrogen bonding and as a secondary dopant increasing the mobility of charge carriers between colloidal particles. The good performance of water resistance was also confirmed again in experiments using artificial sweat (Fig. S8). As a polyol, xylitol is expected to form a hydration film on the surface of the film, preventing moisture (or other water-based reagents from the outside world) from directly penetrating into the film. This helps slow or reduce the impact of moisture on PEDOT: PSS films.

### Evaluating the performance of the PEDOT: PSS electrode

**Fig. 5:**
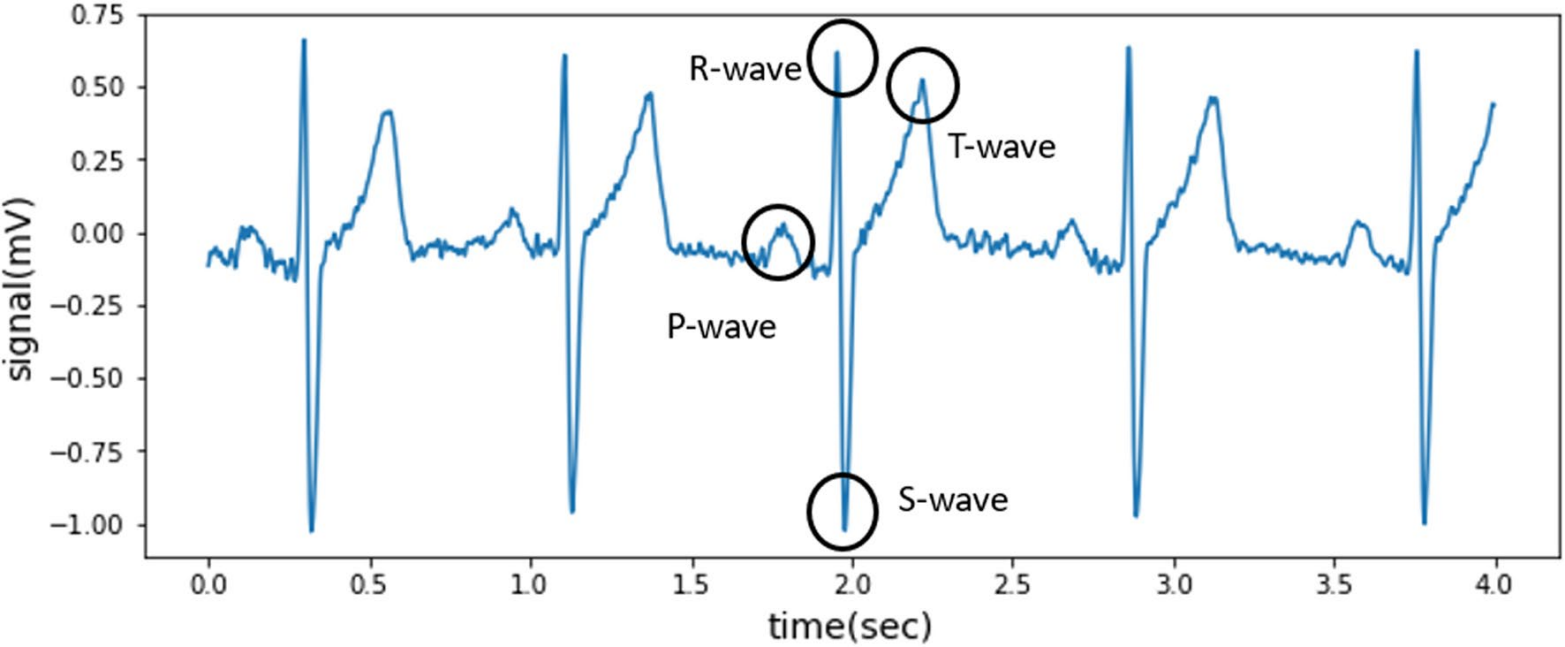
The ECG signals recorded by the PEDOT: PSS electrode. All P-QRS-T morphology is identified

The SNR measured using the PEDOT: PSS electrode was 30.5 dB. The results reveal that the electrode provides effective signal performance. Our results clearly identified P-, T-, and S-waves (Fig. 5).

### Conductivity

Subsequently, we investigated the improvement in the conductivity by adding different concentrations of EG to a PEDOT: PSS/5 wt% xylitol thin film. The concentrations of EG varied from 2 to 5 wt% with an increment of 0.5 wt% in each group. The film electrode with 5 wt% xylitol and 3.5 wt% EG added in PEDOT: PSS had the lowest impedance of 351 Ω, shown in Fig. 4b. The presence of EG may lead to the reduction of gaps between the PEDOT: PSS particles, forming a tighter structure and thereby improving conductivity [53], which is largely affected by the hydrophilicity and dielectric constant of the dopant material used. As the amount of EG increased, the impedance initially decreased and then increased (Fig. S1). When the doping concentration of EG was 4 wt%, the impedance increased. The addition of EG generally reduces the resistive impedance of the PEDOT: PSS films. This is because the presence of EG increases the charge-transfer rate of PEDOT: PSS and reduces the resistive impedance, thereby improving the charge-transfer efficiency between the electrode and material. However, excess EG can increase the resistance of PEDOT: PSS films [54]. Excess EG may decrease the concentration of charge carriers in PEDOT: PSS. This may be because the addition of EG dilutes the conductive component of PEDOT: PSS. In this case, the ability of the charge to transport through the material is reduced, causing the impedance to increase. Different dopants, including EG [55], glycerol (GL) [55], polyglycerol (PG) [55], and butylene glycol (BG) [56], show a tendency to increase and then decrease impedance as the doping level increases. Therefore, it is not necessary to determine the optimum ratio using the step concentration approach. For comparison, we also tested the impedance of the silver and gold electrodes, which were 0.97 Ω and 9 Ω, respectively. Also, for the 3.5 wt% EG/5 wt% xylitol/PEDOT: PSS group, we calculated the sheet resistance and obtained the results as shown in Fig. 4c. As the concentration of EG increased, its impact on the sheet impedance became consistent with the silver electrodes. An appropriate increase in the EG doping concentration reduces the impedance, whereas an excess concentration causes a sharp increase in impedance. Although EG as a solvent optimized the molecular structure of PEDOT, leading to a reduction in the insulating PSS shell and crystallization of PEDOT and the aggregation of PEDOT:PSS particles, macroscopically results in a decrease in impedance [57, 58]. The excess concentration of solvent may cause over-dilution of DEPOT:PSS, causing the aggregation structure to fracture, where the formed pores and cracks would affect structural strength and conductivity. In Tatsuhiro Horii's study, this phenomenon was observed at a concentration of EG at 20% [57]. In this study, the increase in impedance was observed at a concentration of xylitol of 3.5% with EG of 5%. This observation may be related to the addition of xylitol as a solvent, also has an effect on electrical conductivity [59].

We measured and analyzed the skin–electrode contact impedance of the proposed electrodes [54]. The electrodes of the proposed preparation were arranged in the small arm of the tester in the shape of a square of 1 cm^2^, and the skin- electrode contact impedance was measured using an impedance analyzer (Bode 100, OMICRON Lab). The measurements are shown in Fig. 4e, where the dotted line represents the commercial Ag/AgCl electrode impedance data, and the blue line represents the skin impedance data of the proposed electrode. Overall, the skin–electrode impedance was higher than that of the Ag/AgCl electrode from 10 to 103 Hz and slightly lower than that after 2 × 103 Hz. The proposed electrodes, under 1 cm^2^ operating conditions, are in close agreement with the impedance of the current commercial electrodes. The proposed electrode was effective at the macroscopic level. The electrode film can be placed on a steering wheel; when a person holds the electrode (simulating the driving state), the ECG signal can be stably, accurately, and continuously acquired after a brief fluctuation, as shown Fig. 4f.

The mechanisms for the enhancement of the electrical conductivity have been widely studied; phase segregation between PEDOT and PSS by adding EG is currently the most accepted [60]. In experiments on improving conductivity, decreasing thickness, and maintaining a constant transmittance, Crispin et al. [61] and Kim et al. [62] showed that excess insulating PSS was removed from the surface of the film during a post-treatment with an addition of EG, which is a plausible reason for the increase in electrical conductivity. The increasing trend of conductivity is consistent with the results obtained from the experiments in this study.

### Peel-ability

We further investigated the thin-film fabrication process after determining the optimal concentrations of dopants, such as xylitol and EG. Separation is an essential part of thin-film electrode fabrication. We changed the concentration of the PDMS support layer by adding toluene to facilitate d the separation process. The thickness of the PDMS support layer was reduced when using toluene [63, 64], therefore the thin-film electrode separation was easiest when the volume ratio of PDMS to toluene reached 1:1, as shown in Table 1. However, when the toluene volume ratio was increased from 1 to 4, the electrodes became increasingly difficult to detach from the devices, and the morphology deteriorated.

**Table 1:** Ease of peeling and surface properties depending on the ratio of PDMS and toluene Good: ○; Medium: △, Poor: ×

Component ratio	Peelability	Surface quality
10:1	○	○
1:1	△	○
1:2	×	△
1:4	×	×

The peeled-off enhanced PEDOT: PSS electrode is not designed to be directly attached to the human body according to our application scenario but is attached to tools that people often come into contact with in daily activities, such as frequently contacted computer parts (as shown in Fig. 1a). This can enable long-term monitoring of a subject’s ECG signals without affecting normal work.

**Fig. 6:**
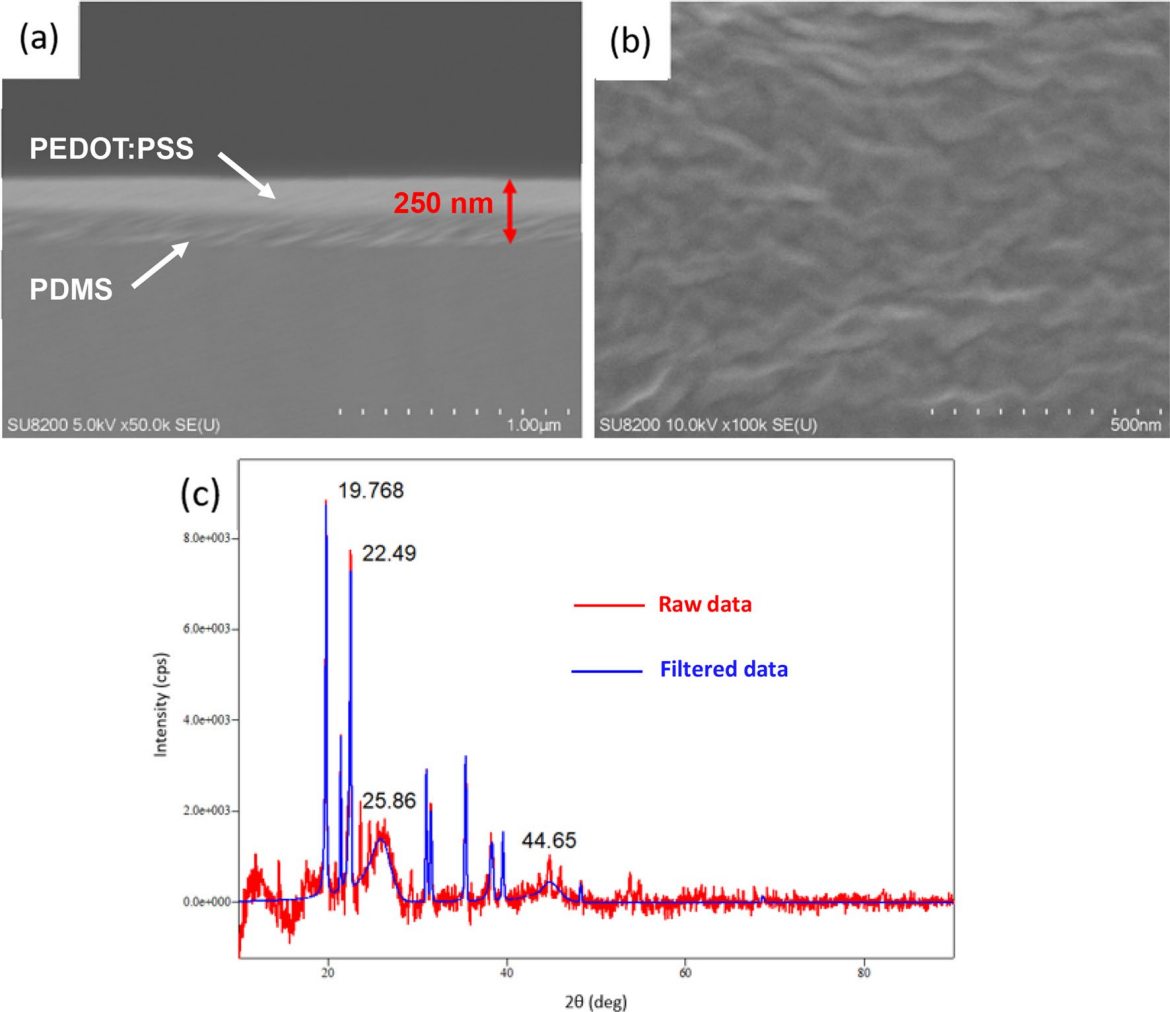
**a**, **b** SEM image of a thin film with a conductive layer of PEDOT: PSS with 5 wt% xylitol and 3.5 wt% ethylene glycol, and a support layer of PDMS diluted 1:1 with toluene. The thickness of the thin film was found to be 250 nm from cross-sectional SEM image. No characteristic shape was found on the electrode surface from the frontal SEM image; **c** AFM results of thin films having conductive layer of PEDOT: PSS with 5 wt% xylitol and 3.5 wt% ethylene glycol and support layer of PDMS diluted 1:1 with toluene. 2θ = 4, 25.86° are the peaks of PEDOT: PSS. The sharp peaks at 2θ = 19.768° and 2θ = 22.49° are the peaks of xylitol

After combining the above three experiments, scanning electron microscopy (SEM) and atomic force microscopy (AFM) analyses were performed on the thin-film electrodes of PEDOT: PSS with toluene-diluted PDMS (1:1) as the support layer with xylitol 5 wt% and EG 3.5 wt%. The results are presented in Fig. 6. The SEM images of a thin film with a conductive layer of PEDOT: PSS with 5 wt% xylitol and 3.5 wt% EG and a support layer of PDMS diluted 1:1 with toluene are shown in Fig. 6a, b. The thickness of the thin film was found to be 250 nm from a cross-sectional SEM image, of which the thickness of the PEDOT: PSS membrane was approximately 115 nm. This is in line with the membrane molding thickness of 90–105 nm under the process conditions reported in the literature [56] (spin-coating speed = 1000 rpm). No sacrificial layer was used to investigate the thickness of the thin films. No characteristic shapes were observed on the electrode surface in the frontal SEM images. The AFM results for the thin film with a conductive layer of PEDOT: PSS with 5 wt% xylitol and 3.5 wt% EG, and a support layer of PDMS diluted 1:1 with toluene are shown in Fig. 6c. Although no match was made in the database because the electrode is a mixture of organic materials and the sample was too thin, it can be seen from Wang et al. [65] that 2θ = 4° (which was not recognized as a peak), and 2θ = 25.86°, which has the most significant integrated intensity, are the peaks of PEDOT: PSS itself. EG was the main component of PEDOT: PSS. EG amplified the PEDOT: PSS peak, as reported by Okuzaki et al. [66]. The sharp peaks at 2θ = 19.768° and 2θ = 22.49° were identified as xylitol peaks, as reported by Negar et al. [67].

### Logical judging circuit design

**Fig. 7:**
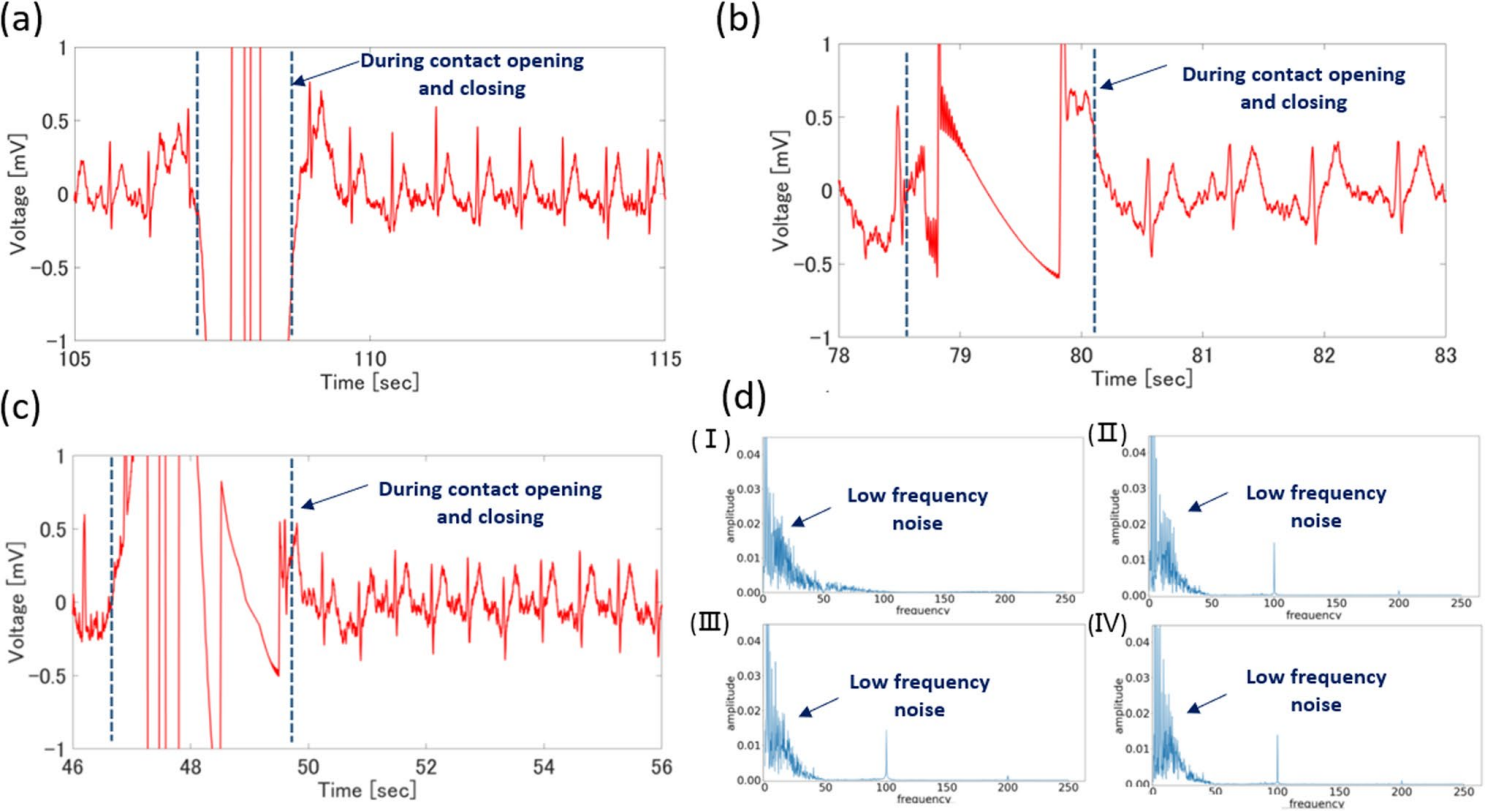
**a** ECG fluctuation when changing different input channels using designed logic circuit. After changing the input channel, the ECG wave return to stability within 3 s; **b** ECG waveform obtained by ECG measurement with USB power supply and commercial Arduino also shows clutter during contact switching, but quickly returns to normal after contact (skin and electrode); **c** The ECG waveform obtained by ECG measurement with DC power supply and commercial Arduino also shows clutter during the contact switching period, but quickly returns to normal after contact (skin and electrode); **d** Picture of fast Fourier transform of each electrocardiogram waveform. Among them (I) is measured by a commercial ECG electrode kit, (II) is measured by an Arduino controlled relay connected to a personal computer, (III) is controlled and measured by an Arduino driven by a battery (direct current), (IV) is measured by the designed logic integrated circuit controls

Because the judging circuit of a commercial microchip is not optimized, significant noise affects the waveform under large-area electrode measurements. In addition, signal input channels are limited to conventional Arduino chips. Thus, we designed a logical judging circuit (for the detailed design process, please refer to the Supplementary Materials) and connected it to the 3.5 wt% EG/5 wt% xylitol/PEDOT: PSS thin-film electrode. We increased the number of input channels in the circuit and successfully achieved selective measurements on the electrode. The condition of selective measurement was introduced previously and only allows connection for the input channel subject to the highest pressure. As a result, as shown in Fig. 7, the noise was successfully reduced during measurement with large-area electrodes and the accuracy of the ECG waveform was optimized. Although the opening and closing of the contact point cause interference in the measured ECG waveform (3 s of noise in Fig. 7a), the measurement returns to normal when contact is re-established. To confirm that the appearance of noise was not caused by defects in the designed logic circuit, we compared the processing conditions of different circuits using the same thin-film electrodes and other experimental conditions. Measurement results via USB power and an Arduino are shown in Fig. 7b. The ECG waveform is measured for 5 s, and the ECG waveform measured from the wrist by clamping the electrode is also shown. In both waveforms, the characteristic waveforms of the electrocardiogram, namely sharp, peaked R and T waves, were observed. Therefore, even when connected through a relay, electrocardiogram measurements can be performed normally. Furthermore, it shows the phenomenon of waveform oscillation when switching the contact points. Figure 7c shows the 10-s waveform of the ECG measured using a DC power supply and an Arduino. At 47 s, this waveform shows a change of contact when the finger is moved from one film to another. As the equivalent waveforms in Fig. 7a, b were obtained, it can be seen that the changes in the power supply of the microcontroller station did not affect the switching circuit of the ECG measurement circuit. Therefore, the measurements can be performed despite real-life interruptions.

When using, the electrocardiogram measured by commercial electrodes, USB-Arduino, DC-Arduino, and the purpose-designed logic integrated circuit to determine and measure contact each was measured within 5 s of the normal electrocardiogram. A fast Fourier transform was performed, and the results are shown in Fig. 7d** (I–IV)**. Although not present in the ECG waveform measured at the clamp pole, strong wavelengths at 100 and 200 Hz were present in the three waveforms measured when making contact judgments. It appears that the inclusion of a contact switch circuit would be more likely to cause external buzzing noise owing to the large number of unsuitable circuit components in an electrocardiograph. Therefore, the designed logic circuit is suitable for long-term ECG signal acquisition.

## Conclusion

In this study, the electrical conductivity and water resistance of PEDOT: PSS electrodes were successfully enhanced by adding EG and xylitol. The conductivity of the PEDOT: PSS electrodes was tested by varying the glycol concentration. By adding 3.5 wt% glycol to PEDOT: PSS with 5 wt% xylitol thin-film electrodes with ideal conductivity and water resistance were fabricated. Finally, by mixing the PDMS support layer with toluene at a ratio of 1:1 and adding 5 wt% of xylitol and 3.5 wt% of EG to PEDOT: PSS, a thickness of 250 nm, a sheet resistance of 410 Ω/sq, and an impedance of 351 Ω were obtained. To reduce noise in ECG measurements, we designed a logic judging circuit that effectively enabled the device to automatically determine and connect the input channel under the highest pressure, while simultaneously shutting down other channels, decreasing noise and improving accuracy.

Supporting information

- The influence trend of increasing gradient concentration of EG on the impedance of PEDOT:PSS (word);
- Hardware design of logic judgment circuit (filtering and noise removal) (word);
- Validity testing of hardware circuits (word);
- Experimental method of the influence of power supply mode on logic judgment circuit (word);
- Effects of adding different concentrations of xylitol on the sweat resistance of PEDOT: PSS thin film with 0 wt%, 2wt%, 3wt%, 4wt%, 5wt% Xylitol (word).

### Supplementary Information

Supplementary file1 (DOCX 1301 kb)

## Data Availability

The data that support the plots within this paper and other findings of this study are available from the corresponding author upon reasonable request.
