# Supplementary material for: A combination of logical judging circuit and water-resistant ultrathin film PEDOT: PSS electrode for noninvasive ECG measurement
Source: Discov Nano. 2024 Mar 14;19(1):45. doi: 10.1186/s11671-024-03988-9 (PMC10940549; doi:10.1186/s11671-024-03988-9)
Supplement: Supplementary file 1 — Supplementary file1 (DOCX 1301 kb) [file 11671_2024_3988_MOESM1_ESM.docx]

**Supplementary Materials**

**A Combination of Logical Judging Circuit and Water-resistant Ultrathin Film PEDOT: PSS Electrode for Noninvasive ECG Measurement**

Kewei Song^a, §^, Kayo Hirose^b, §^, Kioto Niitsu^c^, Tsubasa Sui^a^, Hiroto Kojima^c^, Toshinori Fujie^e,^*, Shinjiro Umezu^a,c,d^ *

*^a^Graduate School of Creative Science and Engineering, Department of Modern Mechanical Engineering, Waseda University, 3-4-1 Okubo, Shinjuku-ku, Tokyo 169-8555, Japan.*

*^b^Anesthesiology and Pain Relief Center, The University of Tokyo Hospital, 7-3-1 Hongo, Bunkyo-ku, Tokyo 113-8655, Japan*

*^c^Graduate School of Advanced Science and Engineering, Department of Integrative Bioscience and Biomedical Engineering, Waseda University, 3-4-1 Okubo, Shinjuku-ku, Tokyo 169-8555, Japan.*

*^d^Department of Modern Mechanical Engineering, Waseda University, 3-4-1 Okubo, Shinjuku-ku, Tokyo 169-8555, Japan.*

*^e^School of Life Science and Technology,* *Tokyo Institute of Technology, B-50, 4259 Nagatsuta-cho, Midori-ku, Yokohama, 226-8501 Japan*

**^§^The authors contributed equally.**

***Corresponding authors:**

Shinjiro Umezu, Professor, E-mail: [umeshin@waseda.jp](mailto:umeshin@waseda.jp)

Toshinori Fujie, Associate Professor, E-mail: [t_fujie@bio.titech.ac.jp](mailto:t_fujie@bio.titech.ac.jp)

**
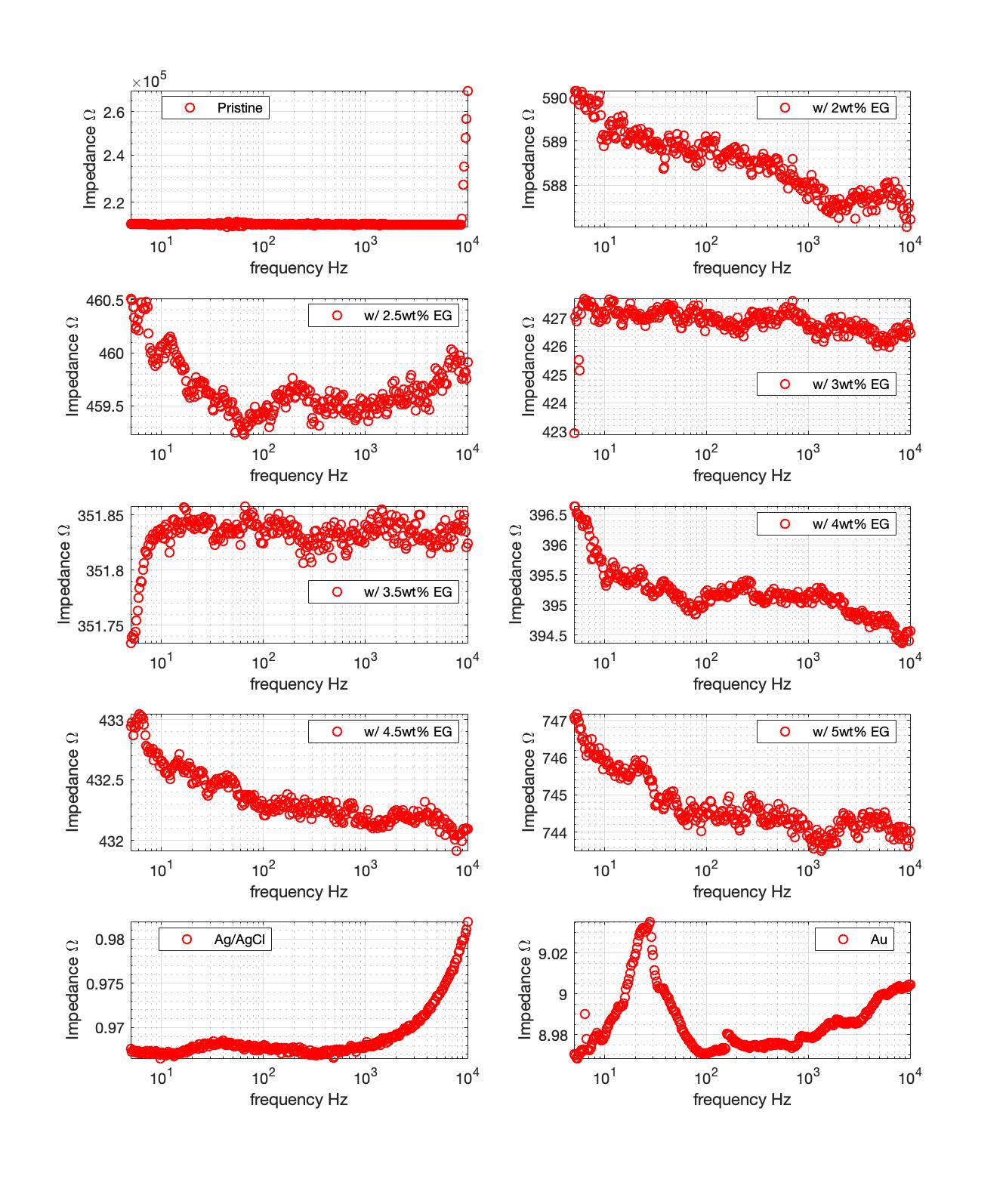
The influence trend of increasing gradient concentration of EG on the impedance of PEDOT:PSS:**

**Figure S1. Impedance when 5wt% of Xylitol and Ethylene Glycol were added to PEDOT: PSS in 0.5wt% increments from 2wt% to 5wt%, and for comparison, the impedance at a silver-silver chloride electrode and a gold thin film electrode.** Measured results. The thin film electrode made by adding 5wt% of Xylitol and 3.5wt% of Ethylene Glycol to PEDOT:PSS had the lowest impedance of 351Ω. Note that the resistance of the silver-silver chloride electrode was 0.97Ω, and the resistance of the gold electrode was 9Ω.

**Hardware design of logic judgment circuit (filtering and noise removal)**

ECG measures the potential difference between two points with the heart as the origin. We built a heart rate monitor that installs on various devices and transmits data to a PC via a mobile battery station. As the electrode, the enhanced PEDOT:PSS electrode prepared in this study was used. In addition to the logic judgment circuit in the manuscript, the functions of the ECG measurement hardware circuit also include filtering and noise removal. The filter circuit diagram is shown in Fig.S2. The left and right potential differences are amplified, a 50Hz notch filter is used to remove white noise, and a high-pass filter reduces the shaking of the baseline, making the whole thing heavy. By combining a low-pass filter, small noise folding is reduced to form a 0.5-30Hz bandpass filter.

To prove the performance of DURTA, only notch filter, notch filter and high pass field were used for ECG examination using RUTA, notch filter and band pass filter respectively.


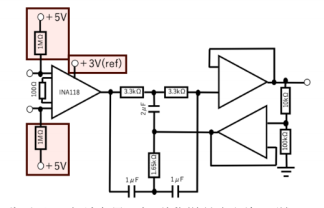
As shown in Fig.S3, the ECG measurement is therefore performed by applying 5 V across both ends and 3 V to the reference electrode before differential amplification. Finally, connect the PC wirelessly using the RN42 shown in Fig.R4 and the Bluetooth module using the above circuit to connect to the circuit for wireless communication. Since the ECG data is 32 bytes per millisecond, which exceeds the 16 bytes of RN42 data transmission, the data is sent in two parts.

**
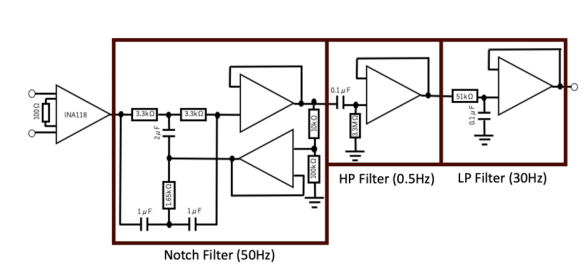
Figure S2. The left and right potential difference amplification circuit of the electrocardiogram measurement circuit is produced.** The notch filter forms a 50 Hz band-pass filter, and the combination of the high-pass filter and the low-pass filter forms a 0.5-30 Hz band-pass filter.

**Figure S3. Circuit that applies voltage to push the baseline higher. Before differential amplification, a voltage of 5 V is applied to both ends and a voltage of 3 V is applied to the reference electrode.**

**
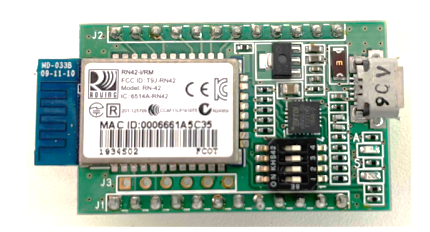
Figure S4. RN42 module, a Bluetooth module used to wirelessly convert electrocardiogram measurement values**.

**Validity testing of hardware circuits**


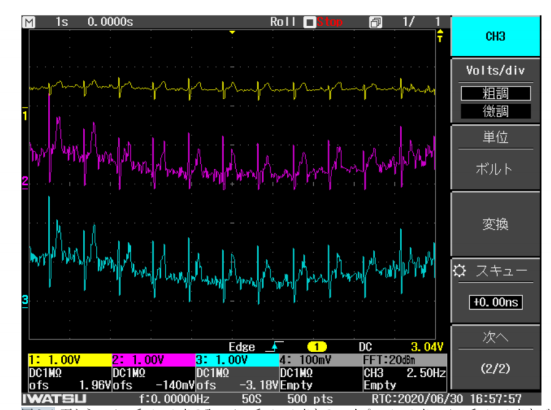
Fig. S5 show the ECG measurements taken by running each filter. Looking at the bottom of the diagram, it is the ECG with only the notch filter, an adaptation of the notch filter and the high pass filter, and the ECG with all the features. There is no difference in waveform between notch filter only and notch filter and high pass filter, and the waveform with all filters working cannot confirm baseline noise or fine noise. In addition, after low-pass filter transmission, the amplitude of the ECG can be found to be reduced.

**Figure S5. ECG measurements taken by running each filter.**


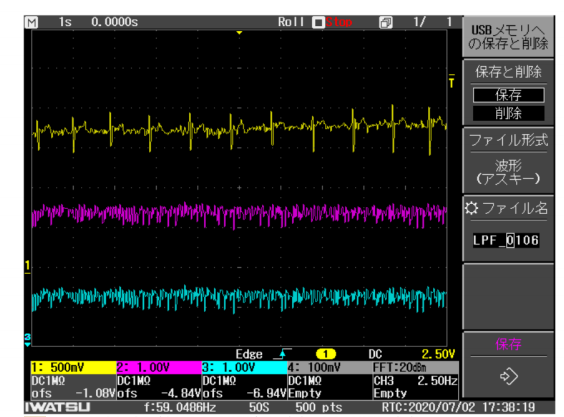
In Fig. S5, starting from the bottom, we test the ECG measurement results using only the notch filter, the high-pass filter with the notch filter, the notch filter and the band-pass filter. There is no difference in waveform between notch filter only and notch filter and high pass filter. Next, Fig. S6 shows the ECG measurement results with the voltage application circuit added to increase the baseline potential. This confirms that the designed hardware circuit can perform stable ECG measurements without touching metal parts such as tables. Figure S7. shows the results of using the RN42 to display the heart on a PC via Bluetooth after transmitting the ECG circuit. It can be confirmed that the electrocardiogram transmitted via Bluetooth can be displayed completely.

**
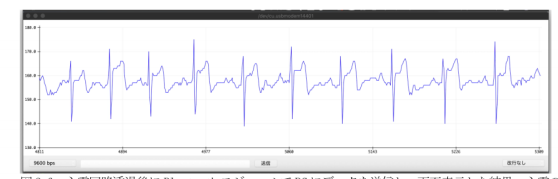
Figure S6. ECG measurement results after adding the voltage application circuit, demonstrating stable ECG measurement**.

**Figure S7. After ECG transmission, the data is sent to the PC via Bluetooth and the results are displayed on the screen.** It can be confirmed that the ECG waveform is not reduced or noise pollution occurs when the ECG is displayed.

**Experimental method of the influence of power supply mode on logic judgment circuit**

**​**We set up three electrodes for measuring ECG on the right hand and three electrodes for measuring on the left hand, for a total of six ECG electrodes. The film used in the electrode is the proposed PEDOT:PSS film. The electrode membrane is connected via relays to the R and L ports of the multifunctional ECG Explorer S00x2. A notched resistive thick film pressure sensor is installed below each electrode.

**Contact judgment during ECG measurements via USB power supply and commercial Arduino:**

The pressure sensor and resistor are connected in series and connected to the USB port of the PC to get an output voltage of 5v from the Arduino UNO universal microcomputer. The voltage between the sensor ports is fed into each of the six analog inputs mounted on the microcomputer board. Since the resistance of this pressure sensor decreases with increasing pressure, the voltage between the sensor ports will be minimum at maximum pressure. Set a condition that at the lowest voltage on the right hand, connect this coil to the membrane located above the pressure sensor, output the voltage to the coil of the relay, and insulate the other membranes from the circuit. The left hand was set up in the same way, sending the potential of the membrane with the highest pressure with each hand to the electrocardiograph. Touch the film with your finger, perform an ECG measurement while switching the film to be contacted, and observe the measured ECG. To compare waveforms, ECG measurements were also performed simultaneously using clip-on electrodes.

**Contact judgment when performing ECG measurements via Arduino using DC power supply:**

Although the electrocardiograph used can transmit data to a personal computer through a wireless connection, in the above environment, the microcomputer and the tooth/cone need to be connected. In this study, the need for ECG measurements in future practical application scenarios (when humans are at work) is considered. Therefore, we tested the method without using PC power supply and tested with DC battery. The contact judgment circuit and the circuit connected to the electrocardiograph are similar to the former, but do not need to be powered by a battery driver or socket. You can use a 9V dry battery to supply power to the power input end of the microcomputer for electrocardiogram measurement and observe the measured electrocardiogram waveform.

**Use the designed logic IC to perform contact judgment during ECG measurement：**

Since a judgment circuit using a general-purpose microcomputer is not the most optimized circuit for this purpose, it may generate unnecessary noise. Additionally, Arduino can only measure a limited number of analog input points. Therefore, a circuit was made through a logic IC and electrocardiogram measurement was performed.

**Effects of adding different concentrations of xylitol on the sweat resistance of PEDOT: PSS thin film with 0 wt%, 2wt%, 3wt%, 4wt%, 5wt% Xylitol:**

**The specific experimental steps are as follows:**

The artificial sweat (PH is 5.5 at 25℃，JIS L-0848-1978)we use is purchased from Hayashiri Industrial Co., Ltd., with analytical purity. We prepared PEDOT: PSS electrodes with xylitol concentrations of 0 wt%, 1 wt%, 2 wt%, 3 wt%, and 4 wt% respectively (Other material components are consistent with those in the manuscript) and placed them in 5 petri dishes containing the same artificial sweat for observation. The immersion experiments of each sample were carried out in the same environment (temperature: 26°C), and the experimental conditions were recorded after 4 hours, 8 hours, 12 hours and 24 hours respectively.

As shown in Figure S8, PEDOT:PSS electrode added with xylitol showed obvious enhanced anti-sweat properties. Figure S8 (a) shows the PEDOT: PSS electrode containing different gradient concentrations (PEDOT: PSS electrode containing 0 wt%, 1 wt%, 2 wt%, 3 wt%, 4 wt% Xylitol) soaked in artificial sweat (initial state). After four hours, there was no peeling or damage. However, after 8 hours (Figure S8 b)), the control group without xylitol still appeared to peel off (that is, the PEDOT: PSS electrode peeled off from the substrate). After about 24 hours (Figure S8 e)), the PEDOT:PSS electrode without xylitol began to be damaged, while the sample with xylitol added gradually began to peel off but did not show damage. Xylitol itself, as a polyol, has good hygroscopicity and is expected to form a hydration film on the surface of the PEDOT: PSS film, preventing moisture from directly penetrating into the film and improving its water or sweat resistance.


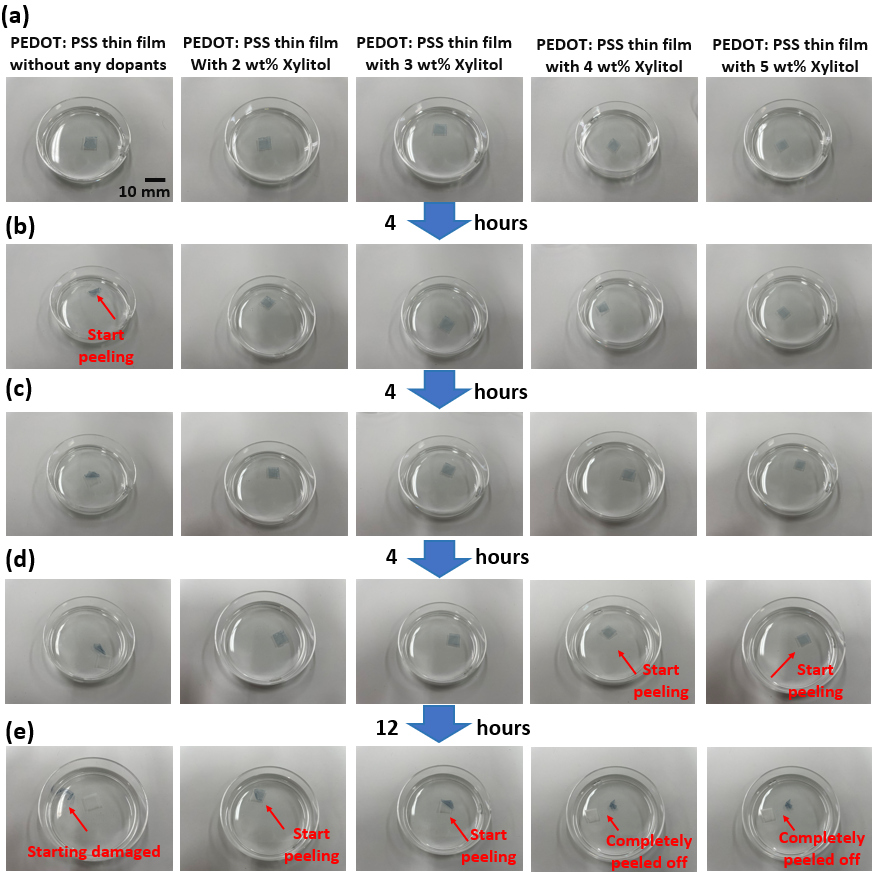
**Figure S8.** **Effects of adding different concentrations of xylitol on the sweat resistance of PEDOT: PSS thin film with 0 wt%, 2wt%, 3wt%, 4wt%, 5wt% Xylitol.** (a) The initial situation when the sample is placed in artificial sweat; (b) The result after the sample is soaked in artificial sweat for 4 hours; (c) The result after the sample is soaked in artificial sweat for 8 hours; (d) The result after the sample is soaked in artificial sweat The results after soaking in artificial sweat for 12 hours; (e) The results after soaking the sample in artificial sweat for 24 hours.
